# Detection of Serum IgG4 Levels in Patients with IgG4-Related Disease and Other Disorders

**DOI:** 10.1371/journal.pone.0124233

**Published:** 2015-04-17

**Authors:** Yuying Su, Wei Sun, Chenqiong Wang, Xuefen Wu, Ye Miao, Hui Xiong, Lin Bai, Lingli Dong

**Affiliations:** 1 Department of Rheumatology and Immunology, Tongji Hospital, Tongji Medical College, Huazhong University of Science and Technology, Wuhan, Hubei, China; 2 Department of Stomatology, Union Hospital, Tongji Medical College, Huazhong University of Science and Technology, Wuhan, Hubei, China; Penn State University, UNITED STATES

## Abstract

**Objective:**

Elevated serum IgG4 levels are an important hallmark for diagnosing IgG4-related disease (IgG4-RD), but can also be observed in other diseases. This study aimed to compare two different testing methods for IgG4: ELISA and nephelometric assay. Both assays were used to measure serum IgG4 concentrations, and to assess the prevalence of high serum IgG4 levels in both IgG4-RD and non-IgG4-RD diseases.

**Methods:**

A total of 80 serum samples were tested using the nephelometric assay and ELISA method that we established. Serum IgG4 concentrations were determined by ELISA for 957 patients with distinct diseases, including 12 cases of IgG4-RD and 945 cases of non-IgG4-RD.

**Results:**

IgG4 levels from 80 selected serum samples examined by ELISA were in agreement with those detected using the nephelometry assay. Meanwhile, the serum IgG4 concentrations measured by ELISA were also consistent with the clinical diagnoses of patients with IgG4-RD during the course of disease. The Elevated levels of serum IgG4 (>1.35 g/L) were detected in all IgG4-RD (12/12) patients, and the prevalence of high IgG4 serum levels was 3.39% in non-IgG4-RD cases. Among them, the positive rates of serum IgG4 were 2.06% in patients with carcinoma and 6.3% in patients with other non-IgG4 autoimmune diseases.

**Conclusion:**

Our established ELISA method is a reliable and convenient technique, which could be extensively used in the clinic to measure serum IgG4 levels. High levels of IgG4 were observed in IgG4-RD. However, this phenomenon could also be observed in other diseases, such as carcinomas and other autoimmune diseases. Thus, a diagnosis of IgG4 disease cannot only be dependent on the detection of elevated serum IgG4 levels.

## Introduction

IgG4-related disease (IgG4-RD) is an idiopathic, fibroinflammatory clinicopathological entity characterized by elevated serum IgG4 levels, tumefaction, and abundant lymphoplasmacytic infiltration of IgG4-positive plasma cells in involved tissues [1], with a middle-aged male predominance. The pancreas, lacrimal gland, salivary gland, lungs, kidneys, skin, and retroperitoneum, virtually every organ, can be affected synchronously and metachronously [2]. The three critical morphological features of IgG4-RD are prominent lymphoplasmacytic infiltration of IgG4-positive plasma cells, storiform fibrosis, and obliterative phlebitis in affected tissues [2–3]. The elevated IgG4 levels in serum and histopathological features are considered to be vitally important hallmarks for the diagnosis of IgG4-RD. Patients with IgG4-RD often present good responsiveness to glucocorticoids, but some individuals relapse during glucocorticoid tapering or withdrawal.

A serum cutoff value of IgG4 levels >1.35 g/L has become widely accepted for the diagnosis of the IgG4-RD [4]. Increased serum IgG4 levels can be observed in most patients with IgG4-RD, and serum IgG4 levels are currently considered to be an important biomarker for IgG4-RD. A ratio of IgG4^+^/IgG^+^ plasmacytes >40% and IgG4-positive plasmacytes >10 per high power field (HPF) in involved tissues contribute to the diagnosis of IgG4-RD [5], especially for patients with a serum IgG4 concentration <1.35g/L. On the other hand, as report goes, serum IgG4 concentrations are elevated in a few patients with pancreatic neoplasia [6], Castleman diseases [7], Churg-Strauss vasculitis etc[8], although the prevalence of elevated IgG4 is significantly lower in these diseases compared with IgG4-RD. Currently,there still remains many unknowns with regard to the clinical implications of serum IgG4 concentrations for the diagnosis of IgG4-RD.Two main methods for measuring serum IgG4 levels have been established, ELISA and nephelometric assays.To date, there are no commercial ELISA kits available in the clinic to determine serum IgG4 concentrations. Our rheumatic laboratory has previously established an ELISA system to detect serum IgG4 levels, which we have successfully used to diagnose patients with biopsy-proven IgG4-RD.

In this study, to validate the effectiveness of our newly established ELISA method, we compared the results conducted by ELISA method and nephelometric assay, and also observed whether high levels of serum IgG4 could be determined in other disorders. We collected 957 serum samples to screen IgG4 concentrations using our newly established ELISA technique. Meanwhile,80 of 957 samples were screened using the nephelometric assay.

## Materials and Methods

### Patients

957 serum samples were collected from patients at Tongji Hospital at Huazhong University of Science and Technology,including cases of IgG4-RD(n = 12), connective diseases(n = 127),nephropathy(n = 36),digestive diseases(n = 60),hematological diseases(n = 38),infectious diseases(n = 18), cardiovascular diseases (n = 25),neurological disorders(n = 24), gynecopathy(n = 11),respiratory disorders (n = 24), carcinoma (n = 535), and other disorders, such as diabetes mellitus, ophthalmopathy, and traumatism (n = 47). There were 12 patients who were eventually diagnosed as IgG4-RD, in accordance with the IgG4-RD diagnostic criteria [9].The study was approved by the ethics committee of Tongji Hospital (Tongji Hospital, Huazhong University of Science and Technology institutional review board approval, IRB ID 20141103). Before the study, written informed consent was obtained from each subject.

### Measurement of serum IgG4 levels

The assays for serum IgG4 detection were performed from March 12, 2013 to July 1, 2014 at the rheumatic laboratory of Tongji hospital, Huazhong University of Science and Technology using our previously established ELISA method [10]. All ELISA experiments were performed in triplicate. A total of 80 of 957 peripheral blood specimens were sent to the clinical laboratory at our hospital to be measured by immunonephelometry(Siemens BN ProSpec automatic protein analyzer) using reagents from Siemens (N Latex IgG4). All assays for IgG4 levels that were above the cutoff value of 1.35 g/L were considered as high IgG4 levels in this study.

### Statistical analysis

Statistical analysis was performed using SPSS version 17.0 statistical analysis software (IBM Corp, New York, NY, USA). Comparisons between paired groups were carried out by the Wilcoxon matched-pairs signed-ranks test. A corrected p<0.05 was considered statistically significant.

## Results

### Comparison of serum IgG4 levels using two different techniques

**Fig 1:**
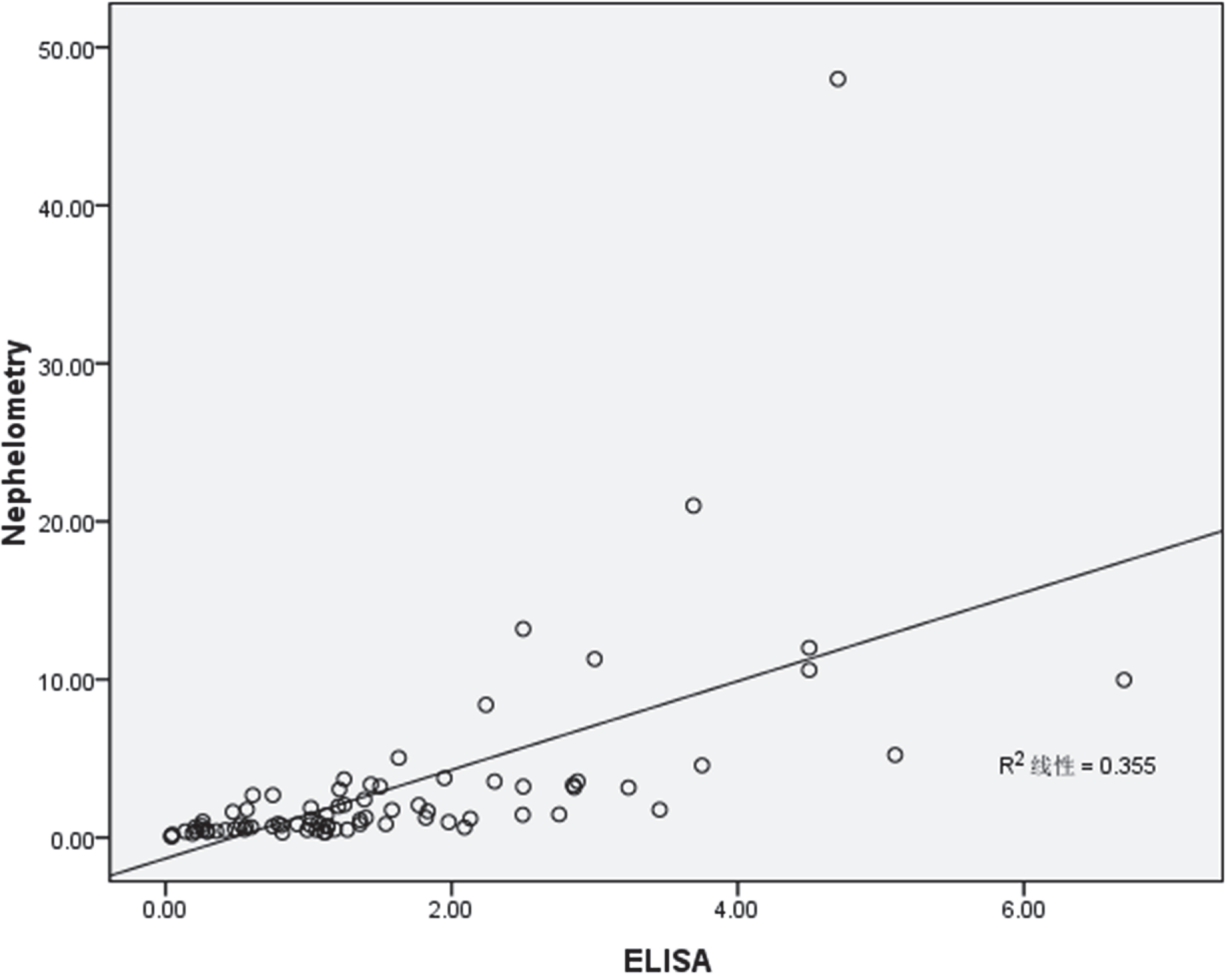
Detection of serum IgG4 levels in 80 patients using both ELISA and nephelometry methods.

A total of 80 of 957 serum samples were detected by both the nephelometric assay and ELISA method. The findings of our established ELISA method agreed well with those of the nephelometric assay (Fig 1). The cost for detecting serum IgG4 levels by the ELISA method was much lower compared with that of the nephelometric assay.

### Serum IgG4 levels in patients with IgG4-RD

**Fig 2:**
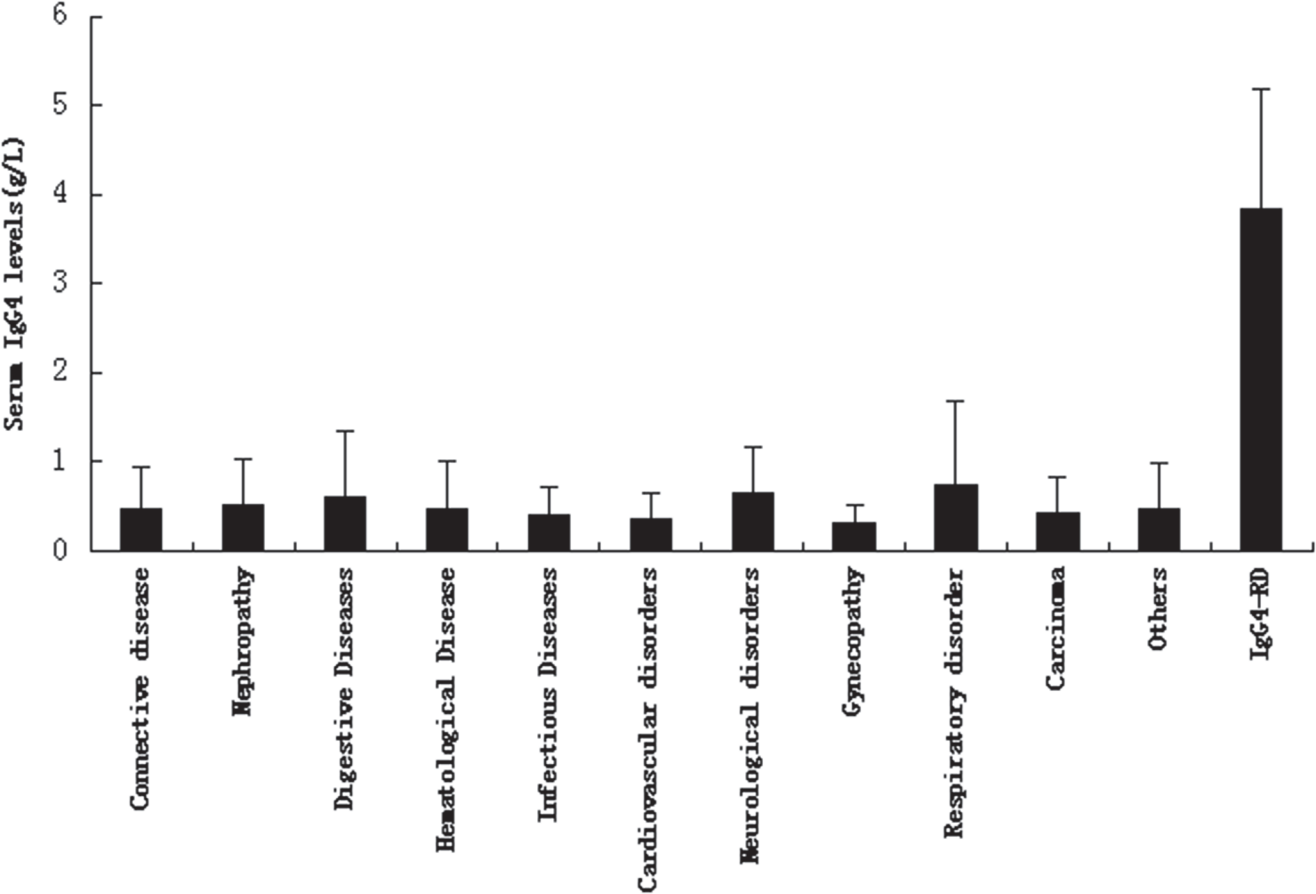
Serum IgG4 levels in various disorders (measured by ELISA). (mean±SD = 0.46±0.49g/L vs. 0.51±0.52g/L vs. 0.61±0.73g/L vs. 0.47±0.53g/L vs. 0.41±0.30g/L vs. 0.36±0.30g/L vs. 0.65±0.52g/L vs. 0.30±0.20g/L vs. 0.73±0.94g/L vs. 0.42±0.41g/L vs. 0.48±0.50g/L vs. 3.83±1.36g/L)

**Fig 3:**
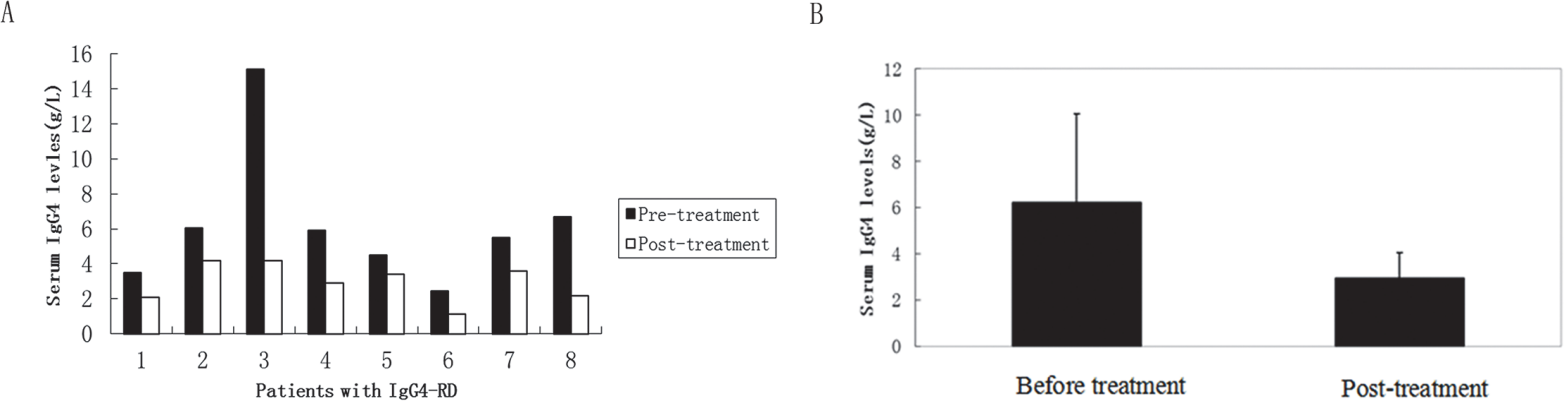
Serum IgG4 levels (mean values) in IgG4-RD patients before and after treatment (measured by ELISA). **A:** Numbers 1 to 8 indicate different patients. Serum IgG4 titers of 8 participants with IgG4-RD were compared before and after treatment. The follow-up time was 1 to 14 months.**B:** A comparison of the mean serum IgG4 levels from 8 IgG4-RD patients before and after treatment. (mean±SD = 6.21±3.86g/L vs. 2.96±1.10g/L). The Wilcoxon matched-pairs signed-ranks test was applied to compare serum IgG4 concentrations; P<0.05.

In this study, serum IgG4 concentrations were significantly higher in IgG4-RD than in other diseases (Fig 2, mean value: 3.83 g/L vs. 0.45 g/L, respectively). In addition,for patients with IgG4-RD,we found that serum IgG4 levels were significantly reduced after at least 1 month of glucocorticosteroid therapy (6.21±3.86g/L vs. 2.96±1.10g/L;for each individual, 0.6–0.8 mg•kg^-1^•d^-1^ was the initial dose of steroid, and the dosage of medications was reduced according to the condition of each patient; Fig 3). These findings indicate that the ELISA method for IgG4 measurement is reliable and that the results obtained using this detection method are consistent with the clinical diagnosis.

### Serum IgG4 levels in non-IgG4-RD

The prevalence of high IgG4 serum levels was 3.39% in non-IgG4-RD when the cutoff value was above 1.35g/L. Elevated levels of IgG4 in the serum (>1.35 g/L) were found in patients with other autoimmune diseases(8/127),nephropathy(2/36), digestive diseases(4/60), hematological diseases(1/38), neurological disorders(2/24), respiratory disorders(3/24), carcinoma(11/535), and other conditions(1/47) (Table 1).

**Table 1:** Patients with elevated levels of serum IgG4 (measured by ELISA).

Final diagnosis	Total cases	Elevated IgG4	Rate	Mean values (g/L)
**Autoimmune diseases**	127	8	6.30%	0.46
**Nephropathy**	36	2	5.56%	0.508
**Digestive diseases**	60	4	6.67%	0.609
**Hematological Diseases**	38	1	2.63%	0.471
**Infectious Diseases**	18	0	0.00%	0.412
**Cardiovascular disorders**	25	0	0.00%	0.361
**Neurological disorders**	24	2	8.33%	0.647
**Gynecopathy**	11	0	0.00%	0.303
**Respiratory disorders**	24	3	1.25%	0.733
**Carcinoma**	535	11	2.06%	0.415
**Others**	47	1	2.13%	0.48
**In total**	945	32	3.39%	0.45

**Fig 4:**
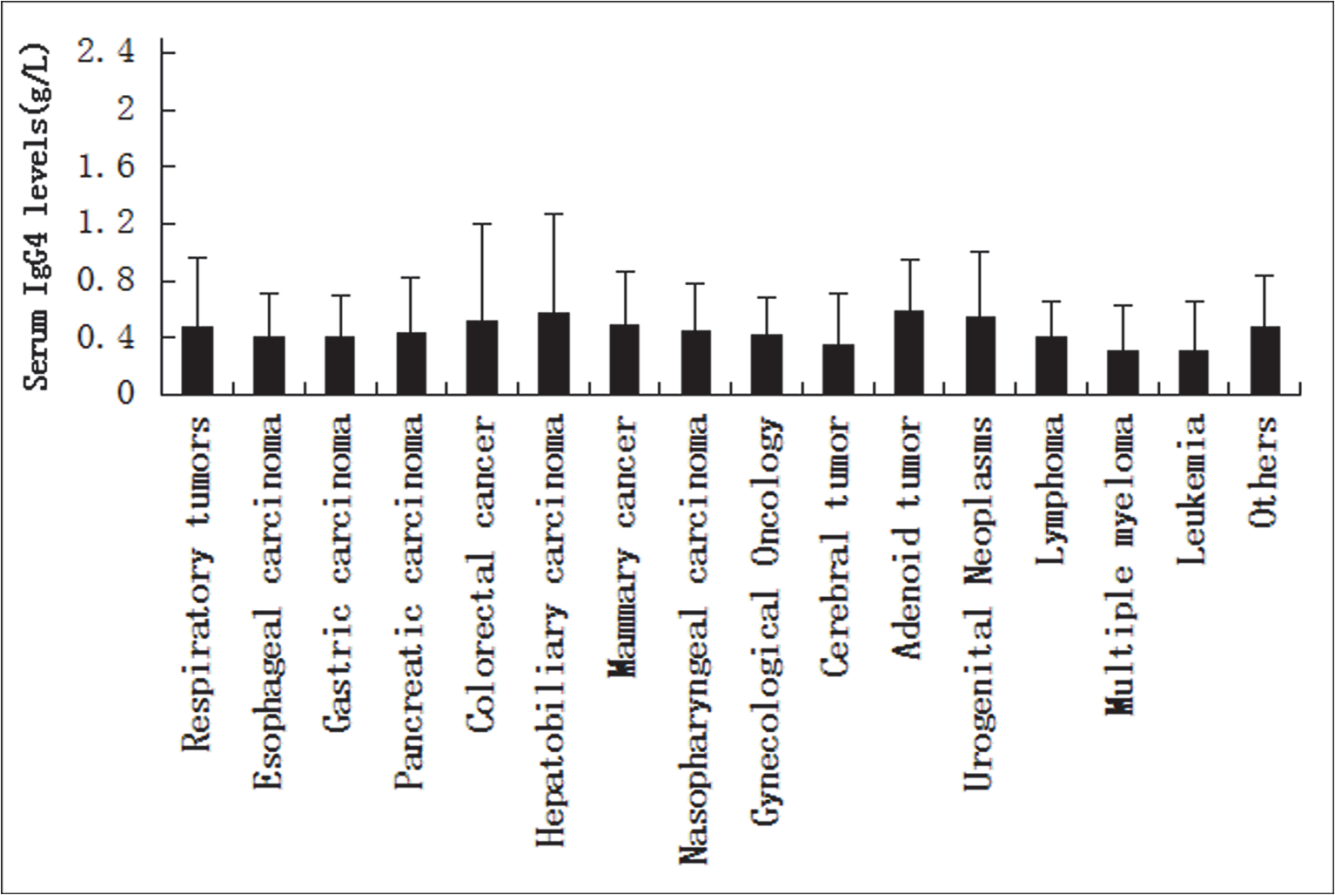
Serum IgG4 levels in patients with different types of tumors. (mean±SD = 0.48±0.49g/L vs. 0.40±0.31g/L vs. 0.40±0.29g/L vs. 0.43±0.39g/L vs. 0.51±0.69g/L vs. 0.57±0.69g/L vs. 0.49±0.38g/L vs. 0.44±0.35g/L vs. 0.42±0.26g/L vs. 0.35±0.37g/L vs. 0.58±0.36g/L vs. 0.55±0.46g/L vs. 0.40±0.25g/L vs. 0.30±0.32g/L vs. 0.31±0.34g/L vs. 0.47±0.37g/L).

Unexpectedly, the highest positive rate of serum IgG4 levels in non-IgG4-RD was 8.33% in neurological disorders,which represented 2 patients with cerebral infarction. The second highest group in this study was 6.67% of patients with digestive diseases, including 3 patients with cirrhosis and 1 with colon polyps. The positive rate of serum IgG4 concentrations in other autoimmune diseases was 6.3%, including 2 patients with ankylosing spondylitis, 2 patients with rheumatoid arthritis, 3 patients with myositis, and 1 patient with suspected connective disease who had elevated serum IgG4 levels (>1.35 g/L). Instead, the positive rate of serum IgG4 in carcinoma was 2.06%, as elevated serum IgG4 levels were determined in 2 lung carcinoma cases (1.39 and 3.84 g/L), 2 rectal carcinoma cases (1.58 and 3.8 g/L), 1 hepatoma case (2.13 g/L), 1 gallbladder carcinoma case (1.63 g/L), 1 cholangiocarcinoma case (2.85 g/L), 1 penile cancer case (1.54 g/L), 2 Acute Myelocytic Leukemia cases (1.36 g/L, 2.49 g/L), and 1 left elbow pleomorphic undifferentiated sarcoma case (1.435 g/L) (Table 2 and Fig 4). Other non-IgG4-RD, such as respiratory disorders and hematological diseases, had a low false positive rate of serum IgG4, ranging from 1.25% to 2.63%. Moreover, no patients with high levels of IgG4 (>1.35 g/L) were found among infectious diseases patients, especially among pulmonary infection and cardiovascular disorder cases (Table 1). Although the cardiovascular system can be involved in IgG4-RD, as reported in the literature, there were no patients with cardiovascular disease who exhibited high serum IgG4 concentrations in our cohort, so we speculate that cardiovascular involvement in IgG4-RD is rare. Our observation of serum IgG4 levels in non-IgG4-RD will help us make a better differential diagnosis in the clinic.

**Table 2:** Elevated levels of serum IgG4 in carcinoma diseases.

Carcinoma category	Number	Mean values (g/L)	Positive cases	Positive rate	Disease
**Respiratory tumors**	93	0.48	2	2.15%	Lung carcinoma
**Esophageal carcinoma**	25	0.40	0	0	No disease
**Gastric carcinoma**	19	0.40	0	0	No disease
**Pancreatic carcinoma**	9	0.43	0	0	No disease
**Colorectal cancer**	33	0.51	2	6.06%	Rectal carcinoma
**Hepatobiliary carcinoma**	25	0.57	3	1.20%	Hepatoma, gallbladder carcinoma, cholangiocarcinoma
**Mammary cancer**	18	0.49	0	0	No disease
**Nasopharyngeal carcinoma**	42	0.44	0	0	No disease
**Gynecological Oncology**	32	0.42	0	0	No disease
**Cerebral tumor**	12	0.35	0	0	No disease
**Adenoid tumor**	6	0.58	0	0	No disease
**Urogenital Neoplasms**	11	0.55	1	9.09%	Penile cancer
**Lymphoma**	70	0.40	0	0	No disease
**Multiple myeloma**	15	0.30	0	0	No disease
**Leukemia**	98	0.31	2	2.04%	Acute Myelocytic Leukemia
**Others**	27	0.47	1	3.70%	The left elbow pleomorphicundifferentiated sarcoma
**In total**	535	0.415	11	2.06%	No disease

## Discussion

Elevated serum levels of IgG4 provide an opportunity to identify IgG4-RD. At present, high serum IgG4 concentrations are recognized as a vitally important diagnostic criterion for IgG4-RD. In addition, it was reported that the serum IgG4 concentrations were associated with the disease activity[11]. To some extent, the detection of serum IgG4 levels after treatment can reflect the therapeutic effects and recurrence of IgG4-RD. Increased levels of serum IgG4 have also been reported in other diseases,for instance, pancreatic carcinoma,cholangiocarcinoma,and sclerosing cholangitis [12, 13].Besides this, a few patients with elevated serum IgG4 levels have been reported who suffered from allergic dermatitis,asthma,and Castleman disease [14]. Nevertheless,about 20–40% of patients with biopsy-confirmed IgG4-RD have been found to have serum IgG4 levels that were within the normal range [15]. Therefore, a revaluation of the detection methods and clinical implications of measuring serum IgG4 levels in IgG4-RD is crucial.

Two techniques to measure serum IgG4 levels have been documented so far: the nephelometric assay and the ELISA method. The former method has been widely used in the clinic. A high hook effect can occur in the nephelometric assay, resulting in false negative outcomes. Furthermore, in the nephelometric assay, test results of serum IgG4 levels can differ when the same reagents are obtained from distinct corporations [16]. However, this phenomenon can be avoided by using the ELISA method. In this study, a correlation was observed between the two methods, the ELISA method and nephelometric assay, for the analysis of 80 serum samples (Fig 1). Furthermore, the serum IgG4 concentrations detected by ELISA method agreed well with the clinical diagnosis of disease. A patient who exhibited high serum IgG4 concentrations detected by the nephelometric assay, but which fell within the normal range when tested using our newly established ELISA method, was ultimately diagnosed not to have biopsy-confirmed IgG4-RD. Moreover, we successfully identified and diagnosed 12 patients with increased serum IgG4 levels by our established ELISA method. In these patients, we found pathological evidence of IgG4-RD and observed that their serum IgG4 levels decreased after treatment (Fig 3).

Those serum samples with IgG4 concentrations >1.35 g/L, which was defined as the cutoff value, showed great sensitivity and specificity (97% and 79.6%, respectively), and a serum ratio of (IgG4/IgG) > 8 demonstrated an increased specificity of 87.5% for IgG4-RD [17]. If a serum IgG4 level >1.44 g/L was used as the cutoff value to diagnose IgG4-RD, the specificity increased to 90.76% [18]. Taiwo N. Ngwa et al. conducted a retrospective study in July 2014 and found that 390 of 6014 patients exhibited high serum IgG4 levels. Among the 390 subjects analyzed, only 39 patients were diagnosed as IgG4-RD, including 10 patients probably with IgG4-RD and 29 subjects definitely with IgG4-RD [19]. They made a conclusion that the false positive rate of serum IgG4 levels, leading to a misdiagnosis of IgG4-RD, was high. Another study indicated that the sensitivity and specificity of serum IgG4 levels for diagnosing IgG4-RD were 90% and 60%, respectively [20]. In our study, 44 of 957 cases exhibited elevated serum measurements above 1.35 g/L, and 12 of 44 patients had definite IgG4-RD. The false positive rate of serum IgG4 levels for the diagnosis of IgG4-RD in our study were not higher than those used by Taiwo N. Ngwa, but this discrepancy might be accounted for by our relatively limited number of samples. Further prospective studies to clarify this issue should be undertaken.Additionally, among 44 subjects, serum IgG4 levels were only slightly above 1.35 g/L. If we raised the cutoff value of serum IgG4 levels to 1.44 g/L, the sensitivity remained unchanged (100%), but the specificity increased to 97.6%. Thus, our established ELISA method could represent a highly sensitive and specific method for the diagnosis of IgG4-RD.

The results of our study illustrated that the positive rate of serum IgG4 levels was 3.39% in non-IgG4-RD patients. We found that 2.06% of carcinoma entities were associated with a positive serum IgG4 titer, including cases of hepatocellular carcinoma, gallbladder carcinoma, lung neoplasms, colorectal cancer,hematologic malignancies,etc (Table 2 and Fig 4). Determining whether any relationship exists between malignant tumors and IgG4-RD will require further study. Pathological findings remain the gold standard for making differential diagnoses between IgG4-RD and cancer.

By contrast, a high positive rate of serum IgG4 was observed in neurological disorders (8.33%) and digestive diseases (6.67%). These positive cases included cerebral infarction (2 cases), cirrhosis (2 cases), and colon polyps (1 case). Additionally, 6.30% of patients with autoimmune diseases exhibited high serum IgG4 levels (>1.35g/L), including 2 patients with ankylosing spondylitis, 2 patients with rheumatoid arthritis, 3 patients with myositis, and 1 patient with suspected connective disease. Based on the medical literature, elevated levels of IgG4 have been found to be associated with liver cirrhosis [21,22] and autoimmune disease, such as rheumatoid arthritis [23] and myositis [22]. The findings of our present study agreed with those previous reports. IgG4-RD encompasses IgG4-related pachymeningitis hypophysis and IgG4-related hypophysitis; however, we observed 2 cases of cerebral infarction with elevated serum IgG4 concentrations. As a rheumatologist, making a differential diagnosis between IgG4-RD and other non-IgG4-RD is a complex decision that is important in the clinic. Thus, until an IgG4-RD diagnosis is finally made, we should pay more attention to the differential diagnosis with non-IgG4-RD, particularly in those pathologies mentioned above, and consider all relevant data: the clinical presentations, serological tests, and principle histopathological changes. The cutoff value (>1.35 g/L) of serum IgG4 for the diagnosis of IgG4-RD is indispensable, but it does not require a diagnosis of IgG4-RD to be made when patients present with serum IgG4 levels >1.35 g/L. Certainly, excluding IgG4-RD is inappropriate for patients with low serum IgG4 levels. The ratio of IgG4^+^/IgG^+^ plasmacytes >40% and IgG4-positive plasmacytes >10 per HPF from pathological assessments are equally important in the diagnosis of IgG4-RD.

Taken together, our newly established ELISA method is convenient and relatively stable for detecting serum IgG4 concentrations, and can be widely used in the clinic. It makes sense only when serum IgG4 level is much higher than 1.35g/L for the diagnosis of IgG4-RD. Differential diagnoses should be made for suspected IgG4-RD cases with high serum IgG4 titers.

## Conclusions

Our newly established ELISA technique can be used in the clinic to measure serum IgG4 levels. Elevated serum IgG4 concentrations can be observed in conditions other than IgG4-RD.
